# Hepatocyte-specific regulation of autophagy and inflammasome activation via MyD88 during lethal *Ehrlichia* infection

**DOI:** 10.3389/fimmu.2023.1212167

**Published:** 2023-11-07

**Authors:** Omid Teymournejad, Aditya Kumar Sharma, Mohammed Abdelwahed, Muhamuda Kader, Ibrahim Ahmed, Hoda Elkafas, Nahed Ismail

**Affiliations:** ^1^ Department of Pathology, College of Medicine, University of Illinois at Chicago, Chicago, IL, United States; ^2^ Hofstra School of Medicine, North Well Health, New York, NY, United States; ^3^ Department of Pathology, School of Medicine, University of Pittsburgh, Pittsburgh, PA, United States

**Keywords:** *Ehrlichia* spp., MyD88, inflammasome, autophagy, HMGB1

## Abstract

Hepatocytes play a crucial role in host response to infection. *Ehrlichia* is an obligate intracellular bacterium that causes potentially life-threatening human monocytic ehrlichiosis (HME) characterized by an initial liver injury followed by sepsis and multi-organ failure. We previously showed that infection with highly virulent *Ehrlichia japonica* (*E. japonica*) induces liver damage and fatal ehrlichiosis in mice via deleterious MyD88-dependent activation of CASP11 and inhibition of autophagy in macrophage. While macrophages are major target cells for *Ehrlichia*, the role of hepatocytes (HCs) in ehrlichiosis remains unclear. We investigated here the role of MyD88 signaling in HCs during infection with *E. japonica* using primary cells from wild-type (WT) and MyD88^-/-^ mice, along with pharmacologic inhibitors of MyD88 in a murine HC cell line. Similar to macrophages, MyD88 signaling in infected HCs led to deleterious CASP11 activation, cleavage of Gasdermin D, secretion of high mobility group box 1, IL-6 production, and inflammatory cell death, while controlling bacterial replication. Unlike macrophages, MyD88 signaling in *Ehrlichia*-infected HCs attenuated CASP1 activation but activated CASP3. Mechanistically, active CASP1/canonical inflammasome pathway negatively regulated the activation of CASP3 in infected MyD88^-/-^ HCs. Further, MyD88 promoted autophagy induction in HCs, which was surprisingly associated with the activation of the mammalian target of rapamycin complex 1 (mTORC1), a known negative regulator of autophagy. Pharmacologic blocking mTORC1 activation in *E. japonica*-infected WT, but not infected MyD88^-/-^ HCs, resulted in significant induction of autophagy, suggesting that MyD88 promotes autophagy during *Ehrlichia* infection not only in an mTORC1-indpenedent manner, but also abrogates mTORC1-mediated inhibition of autophagy in HCs. In conclusion, this study demonstrates that hepatocyte-specific regulation of autophagy and inflammasome pathway via MyD88 is distinct than MyD88 signaling in macrophages during fatal ehrlichiosis. Understanding hepatocyte-specific signaling is critical for the development of new therapeutics against liver-targeting pathogens such as *Ehrlichia*.

## Introduction

*Ehrlichia* are tick-borne obligate intracellular Gram-negative bacteria that reside in endosomal compartments, primarily infecting monocytes, and macrophages (1–3). Unlike other Gram-negative bacteria, *Ehrlichia* lacks peptidoglycan and lipopolysaccharide (LPS). *Ehrlichia* species causes Human Monocytic Ehrlichiosis (HME), a life-threatening acute febrile illness characterized by leukopenia, thrombocytopenia, and elevated liver transaminases (1, 4, 5). If not treated early in infection, patients develop several complications including liver failure and sepsis followed by multi-organ failure (5–7).

*E. chaffeesnis* is the major cause of HME; however, this species causes abortive infection in immunocompetent wild type (WT) mice. *E. japonica* is an adapted murine bacterial pathogen that is closely related to *E. cahffeensis* (5, 7, 8). We have previously reported that infection of WT mice with virulent *E. japonica* (previously called *Ixodes Ovatus Ehrlichia*), causes severe and fatal disease in wild type mice. This murine model of fatal ehrlichiosis recapitulates the immunopathologic and laboratory findings in patients with severe and fatal HME. Similar to *E. chaffeensis*, *E. japonica* infects reticuloendothelial system causing disseminated infection. However, these bacteria primarily target liver, where patients present initially flu-like symptoms as well as liver dysfunction followed by sepsis and multi-organ failure if untreated appropriately. Fatal *E. japonica* infection triggers activation of canonical and non-canonical inflammasome pathways mediated by CASP1 and CASP11, respectively in macrophages (5, 9). Although *Ehrlichia* is a Gram-negative bacterium, it lacks LPS, a major PAMP that activates the canonical and non-canonical inflammasome pathways during infections with other Gram-negative bacteria (7). Additionally, unlike other Gram-negative bacteria such as *Rickettsia*, *Ehrlichia* resides intracellularly within a Phagosome and do not escape into the cytosol (10). Nonetheless, CASP1 activation in macrophages during fatal ehrlichiosis leads to the release of interleukin (IL)-1β and IL-18 in the liver. Hepatic IL-18 triggers the induction of pathogenic adaptive immune responses and excessive inflammation, which contribute to liver injury (5, 11–13). On the other hand, activation of the non-canonical pathway following severe and fatal *Ehrlichia* infection leads to pyroptosis of macrophages, a form of inflammatory cell death, and systemic secretion of high mobility group complex B1 (HMGB1) (7, 14). HMGB1 acts as an endogenous damage-associated molecular pattern (DAMP) that triggers the innate immune response via binding to toll-like receptor 2 (TLR2), TLR4, and TLR9, which contributes to infection-induced sepsis caused by several pathogens (9, 15–19). Binding of HMGB1 to the receptor for advanced glycation end products (RAGE) also triggers activation of CASP1 and canonical inflammasome pathways in macrophages, which further promotes inflammation and cell death (14, 20, 21).

Our prior studies demonstrated that MyD88 signaling in macrophages is a crucial pathway that mediates liver injury during lethal *Ehrlichia* infection in mice by activating the deleterious non-canonical inflammasome pathway (5, 6, 9). We have recently showed that *Ehrlichia* infect and replicate in hepatocytes (HCs) (7). In this study, we examined the role of MyD88 in the regulation of inflammasomes and autophagy in HCs. Our data suggested a dual protective and pathogenic role of MyD88 in HCs.

## Materials and methods

### Ethics statement

This study was conducted according to the principles of the Declaration of Helsinki and under ethical guidelines from the University of Pittsburgh and the University of Illinois at Chicago, Institutional Review Boards. All animal studies and procedures were conducted in strict accordance with the recommendations in the Guide for the Care and Use of Laboratory Animals of the National Institutes of Health’s Office of Laboratory Animal Welfare (Assurance Number A3187-01). The Divisions of Laboratory Animal Resources at the University of Pittsburgh and the University of Illinois at Chicago are accredited by the American Association for the Assessment and Accreditation of Lab Animal Care (AAALAC). The Institutional Animal Care and Use Committees at the University of Pittsburgh and the University of Illinois at Chicago approved the protocol.

### Mice and *Ehrlichia* species

Female 7- to 8-week-old C57BL/6 mice deficient in MyD88 (MyD88^-/-^) and wild type (WT) mice were obtained from Jackson Laboratory (Bar Harbor, ME). All mice were maintained in a pathogen-free environment and were observed daily for signs of illness and survival. The highly virulent monocytic *E. japonica* strains or mildly virulent *Ehrlichia muris* (EM) were used in this study and were provided by Dr. Yasuko Rikihisa (Ohio State University, Columbus, OH).

### Preparation of *Ehrlichia* inoculum

Purified, cell-free *E. japonica* or EM organisms were prepared from spleens and livers of *E. japonica*- or *E. muris*-infected mice harvested on day 7 post-infection (p.i.). following a series of sonication and centrifugation steps. Purified bacteria were suspended in sucrose phosphate buffer as described previously (22, 23).

### Isolation of primary murine hepatocytes and murine hepatocyte cell line

HCs were isolated from naive or infected WT and MyD88^-/-^ mice by perfusion technique as described (9). The purity of the isolated primary HCs was evaluated as described previously (24). Isolated primary HCs (4×10^5^ cells/ml) were then plated on 0.2% gelatin-coated 6-well culture plates. In certain experiments, we used the murine HC cell line AML2 (CRL-5564, ATCC).

### *In vitro Ehrlichia* infection and treatment of murine primary hepatocytes or hepatocyte cell lines

*E. japonica* was added to cultured primary murine HCs or AML2 HCs at a multiplicity of infection (MOI) of 5 before infection, cells were pretreated for 1 hr. with the MyD88 inhibitor ST 2825 (APExBIO Technology LLC, Houston, TX) at a 15 μM to block MyD88 signaling. ST 2825 is a specific inhibitor of MyD88 dimerization that inhibits the homodimerization of the MyD88 Toll-interleukin receptor (TIR) domains without affecting the homodimerization of the death domains. TIR domain inhibition interferes with the recruitment of interleukin receptor-associated kinase 1 (IRAK1) and IRAK4 by MyD88. In our initial standardization experiments, we used several doses of MyD88 inhibitor, as suggested by other studies (25, 26) and examined the efficacy of inhibition on MyD88 expression as well as viability of the cells. In all subsequent experiments, we treated cells with 15 μM of the inhibitor, as this dose was able to inhibit MyD88 signal without affecting the viability of the cells (**Supplementary Figure 1**). The effect of MyD88 inhibitor is specific as treatment of cells with other inhibitors such as CASP1 inhibitor did not result in similar effect seen by the MyD88 inhibitor. To block CASP1 or CASP11 activation, infected cells were pretreated with 30 μg/mL CASP1 (Ac-YVAD-cmk, *invivoGene*) or 40 μM CASP11 inhibitors (Wedelolactone; Santa Cruz). To block mTORC1 activation, infected cells were pretreated with 10 µM mTORC1 inhibitor rapamycin (*InvivoGen*, San Diego, CA) for 30 min followed by infection or fresh media control. Uninfected control cells were cultured with mock antigen (antigens prepared from lysed uninfected splenocytes or liver cells). All cells were collected 24 hr p.i. for further analysis by immunoblot, and supernatants were collected and stored at minus 80°C for cytokine analysis.

### Cell viability assay

The MTT assay (ab211091, Abcam, Cambridge, UK) was employed to assess cell viability following treatment with the MyD88 inhibitor ST 2825 (15 μM) at various time points. HCs were pre-treated with the MyD88 inhibitor ST 2825 for different durations (2, 4, 12, and 24 hours) and subsequently plated into 96-well plates. After treatment, each well received 50 μL of serum-free media and 50 μL of MTT reagent, and the plate was incubated at 37°C for 3 hours. Following incubation, 150 μL of MTT solvent was added, and the plate was incubated for 15 minutes. The absorbance was measured at 590 nm, and the percentage of cytotoxicity in treated cells was determined in accordance with the manufacturer’s instructions.

### Measurement of bacterial number using quantitative real-time PCR

The number of *E. japonica* in infected hepatocytes was determined at 24 hr p.i. by quantitative reverse transcription PCR (RT-qPCR) of the *Ehrlichia dsb* gene as described previously (11). Primers and probes were used (**Supplementary Table 1**). The absolute copy number of *Ehrlichia* was determined based on serial dilutions of plasmids containing the *Ehrlichia dsb* gene. Results were expressed as *Ehrlichia* copies per ng of DNA. The number of live replicating bacteria was determined by RT-qPCR analysis of the 16s rRNA as described previously (11).

### Immunoblot analysis

HCs were lysed in RIPA buffer (Thermo Fisher Scientific, Waltham, MA) supplemented with protease inhibitors and 1 mM phenylmethyl sulphonyl fluoride (PMSF). The total protein content of the lysates was measured using a Bicinchoninic Acid Assay Kit (Pierce Biotechnology, Waltham, MA). According to standard protocols, Membranes were processed and probed with the following primary antibodies: anti– CASP1 (1:100; EMD Millipore, Billerica, MA), anti- CASP11 (1:100, Cell Signaling), anti- CASP3 (1:100, Cell Signaling), anti-LC3-II (1:1000; Sigma-Aldrich, St. Louis, MO) and anti-p62 (SQSTMI) (1:1000; Cell Signaling). Anti-phospho S6 was from Cell Signaling Technology, Inc. (Danvers, MA) and used at a 1:1000 dilution. Blots were washed in TBST and probed with peroxidase-conjugated bovine anti-rabbit secondary antibodies (1:10000; Santa Cruz Biotechnology, Santa Cruz, CA). Specific signals were developed using the ECL-Plus system (GE Healthcare, Chicago, IL). Blots were stripped with Restore Western Blot Stripping Buffer (Pierce) and re-probed with anti-GAPDH (1:1000, Cell Signaling Technology, Inc.) or anti-β-actin (1:1000, Cell Signaling) as loading controls. The density of bands in the blots was determined using ImageJ software version 1.51W (NIH, Bethesda, MD). LC3II was determined by normalization to GAPDH.

### RNA extraction and RT-qPCR

Total RNA from HCs was isolated using Trizol^™^ Reagent (Invitrogen Life Technologies, Carlsbad, CA). For cDNA synthesis, 2 μg of total RNA was reverse transcribed in a final volume of 20 μl using the iScript™ reverse transcription supermix kit (Bio-Rad, Hercules, CA), as specified by the manufacturer. In a Bio-Rad T100 PCR/Thermal Cycler, cDNA was combined with specific primer sets (**Table S1**) using SsoAdvanced™ Universal SYBR^®^ Green Supermix (Bio-Rad).

### Cytokine measurement and HMGB1 ELISA

The mouse cytokine array C1000 (RayBiotech, Peachtree Corners, GA) was used to detect multiple cytokines in the HCs culture supernatant. HCs (5x10^5^ cells) were cultured in 6-well plates at 37°C for 48 hr. and 5% CO_2._ According to the manufacturer’s recommendations, culture supernatants were collected from naive and infected cells at 24 hr p.i. and processed for use in the array. Briefly, membranes were incubated in the blocking buffer at room temperature for 30 min, followed by washing, adding culture supernatant, and incubating at 4°C overnight. The membranes were then washed three times using a wash buffer and then incubated with biotin-conjugated antibodies at room temperature for 2 hr. Finally, membranes were washed and incubated with horseradish peroxidase-conjugated streptavidin at room temperature for 2 hr. Secreted proteins were detected using a luminescence detector (Bio-Rad), and the spots were quantified by Image J. Background staining was subtracted, and the signal was normalized to positive controls on each membrane to obtain relative protein concentrations, represented as fold change compared to naïve (uninfected) and untreated HCs. The concentration of HMGB1 released into the culture supernatant was determined using the mouse HMG1/HMGB1 ELISA Kit (lot # 116578, LifeSpan Biosciences).

### Immunofluorescence staining and confocal microscopy

Staining HMGB1 aggregates, and quantification by confocal microscopy were performed as previously described (7). Briefly, HCs were cultured on coverslips infected with *E. japonica* at MOI of 5, washed three times with PBS, fixed with 2% paraformaldehyde for 20 min, and permeabilized with 0.1% Triton X-100 in PBS for 30 min. After blocking with 5% BSA (A2153, Sigma-Aldrich) for 60 min, the following primary antibodies were added for 2 hr. at room temperature: rabbit anti-HMGB1 (1:100, Cell Signaling Technology, Inc.) and anti-p62 (Cell Signaling 1:100). Next, cells were washed and then incubated with fluorescently labeled anti-rabbit secondary antibody DyLight (1:500, VectaFluor, Vector Labs, Burlingame, CA) for 1 hr. Finally, nuclei were stained with DAPI, and cells were analyzed by confocal microscopy (Olympus Fluoview 1000).

### Statistical analysis

The two-tailed *t-*test was used for comparison of mean values for two experimental groups, and a two-way ANOVA (analysis of variance) followed by *post hoc* testing using Tukey’s Honestly Significant Difference (HSD) method for comparisons of multiple experimental groups. Data were expressed as mean ± SD. P ≤ 0.05, P ≤ 0.01 and P ≤ 0.001.

## Results

### The TLR9-MYD88 axis is differentially upregulated in HCs following infection with virulent *Ehrlichia*

Innate immune responses to intracellular pathogens are triggered by TLR signaling via the adaptor molecules MyD88 or TRIF (27, 28). To examine how innate immune response in HCs senses *Ehrlichia*, we analyzed the differential expression of mRNA of several TLRs, MyD88, and TRIF in an HC cell line infected with either mildly virulent EM or highly virulent *E. japonica*. Our data show that *E. japonica* infection of HCs induced significant upregulation of MyD88, TLR9 and TRIF (*P<0.05, **P<0.01, ***P<0.001, respectively) when compared to EM infection of HCs (**Supplementary Figures 2A–C**). Although there was upregulation of TLR2 and TLR7 in both *E. muris* and *E. japonica*-infected primary HCs compared to naïve cells, we did not detect a significant difference in mRNA expression of TLR2 and TLR7 **(Supplementary Figures 2D, E**). These data are consistent with our previous data showing that the TLR9-MyD88 axis is likely the major pathway that regulates the innate immune response to *Ehrlichia* infection in macrophages, the primary target cells for *Ehrlichia* (5).

### MyD88 signaling controls intracellular survival and replication of *Ehrlichia* in hepatocytes

**Figure 1:**
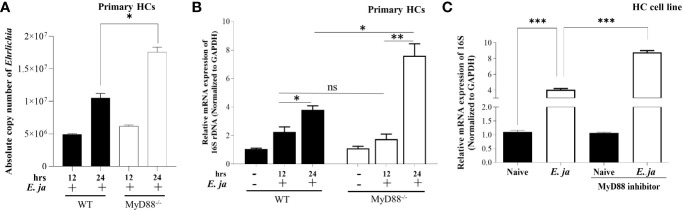
*Ehrlichia* replication in hepatocytes is regulated by MyD88. **(A)** Absolute copy number of intracellular bacteria in primary *E japonica*-infected WT and MyD88^-/-^ HCs at 12 and 24 hr p.i. via amplification of *dsb* gene. **(B)** Relative expression of *E japonica* 16s rRNA normalized to GAPDH to determine live replicative *Ehrlichia* in primary WT and MyD88^-/-^ HCs in the presence/absence of *E japonica* infection at 12 and 24 hr p.i. **(C)** Relative expression of 16s rRNA, normalized to GAPDH, in uninfected and *E japonica*-infected HCs in the presence/absence of MyD88 inhibitor at 24 hr p.i. Data are presented as Mean ± SD from three different experiments. (*P<0.05, **P<0.01, ***P<0.001).

To investigate the role of MyD88 in the pathogenesis of *Ehrlichia*-induced liver injury, we infected WT and MyD88^-/-^ primary HCs with *E. japonica* at an MOI of 5 and measured the number of intracellular bacteria by PCR. We measured the absolute copy number of *E. japonica* organisms in primary HCs at 12 hr. and 24 hr p.i. via amplification of *dsb* gene. We found that the number of *E. japonica* in both WT and MyD88^-/-^ HCs was significantly higher 24 hr p.i. (*P<0.05) compared to 12 hr. p.i. However, the numbers of *Ehrlichia* were dramatically higher in MyD88^-/-^ HCs at 24 hr p.i. than WT-HCs at the same time point (**Figure 1A**). To determine whether the intracellular bacteria measured by *dsb*-based PCR are measuring live, but not dead bacteria, we also analyzed the number of intracellular bacteria by 16S rRNA as described under M&M. As shown in previous studies by us and other investigators (5, 29), there is a strong correlation between the bacterial 16S rRNA and viability of *Ehrlichia*. To determine if the difference in the bacterial number was due to a difference in survival and/or replication of live *Ehrlichia*, we measured the expression level of *Ehrlichia* 16S rRNA by RT-qPCR at two time points 12 hr and 24 hr p.i. To this end, cells were infected with *E. japonica*. At 4hr post infection, the extracellular bacteria were washed and infected cells were further incubated up to 24 hr. At 12 hr p.i., there were significantly higher numbers of intracellular *Ehrlichia* within WT- and MyD88^-/-^ HCs compared to uninfected cells; however, there were no significant differences between the two infected groups. On the other hand, although bacterial number was higher in both WT- and MyD88^-/-^ HCs at 24 hr compared to 12 hr, we detected a significant (*P<0.05) increase in the number of intracellular *Ehrlichia* in MyD88^-/-^ HCs compared to infected WT-HC, suggesting a higher replication of live bacteria in HCs lacking MyD88 signals (**Figure 1B**). To confirm the data in primary HC from KO mice, which may have compensatory mechanisms, we also measured the number of intracellular bacteria in HC cell line treated with MyD88 inhibitor. The findings in primary HCs were consistent with data from *E. japonica*-infected HC cell line (AML2 HCs) treated with MyD88 inhibitor. Lack of MyD88 signaling in infected HCs treated with MyD88 inhibitor, as described under M&M, resulted in a higher number of intracellular live *Ehrlichia* compared to similarly infected but untreated AML2 cells at 24 hr p.i. (**Figure 1C**). The consistency between data obtained from MyD88^-/-^ HCs as well as HC cell line treated with MyD88 inhibitor suggest specificity of the MyD88 inhibition. Additionally, our standardization experiments using other inhibitors such as CASP1 inhibitor did not recapitulate the effect of MyD88^-/-^ HCs as shown below. Together, these data suggest that MyD88 signaling plays a host-protective mechanism by controlling intracellular bacterial survival and/or replication in infected HCs.

### MyD88 differentially regulates canonical and non-canonical inflammasome pathways in HCs during infection with virulent *Ehrlichia*

**Figure 2:**
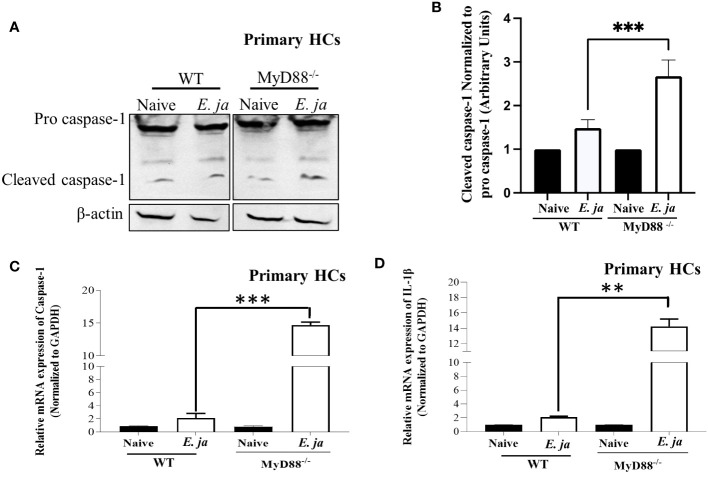
CASP1, in HCs, is negatively regulated by MyD88 in fatal infection. **(A, B)** Immunoblot of pro and cleaved CASP1 in uninfected and *E japonica*-infected primary WT and MyD88^-/-^ HCs at 24 hr p.i. **(C)** Relative mRNA expression of *Casp1* in uninfected and infected primary WT and MyD88^-/-^ HCs at 24 hr p.i. **(D)** Relative mRNA expression of *IL-1β* in uninfected and infected primary WT and MyD88-inhibited HCs at 24 hr p.i. Data is from three independent experiments represented as Mean ± SD (**P<0.01, ***P<0.001).

**Figure 3:**
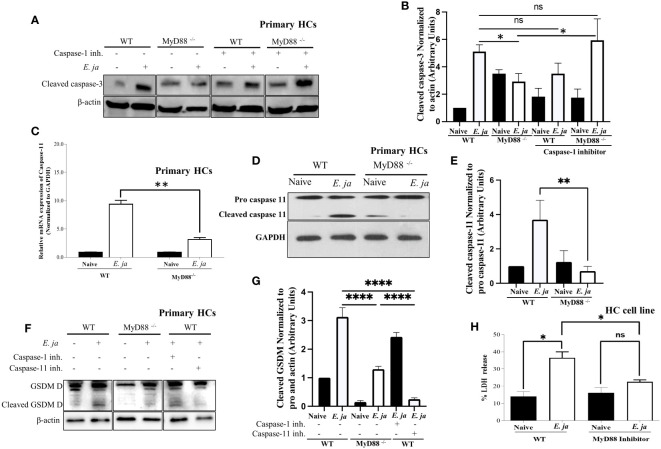
CASP 3 and CASP11, in HCs, is positively regulated by MyD88 in fatal infection. **(A, B)** Immunoblot analysis of cleaved CASP3 protein at 24 hr p.i., normalized to β-actin, in uninfected and *E japonica*-infected primary WT and MyD88^-/-^ HCs cultured in the presence or absence of CASP1 inhibitor. **(C)** Relative mRNA expression of active CASP11 production in uninfected and *E japonica*-infected primary WT and MyD88-inhibited HCs at 24 hr p.i. **(D, E)** Immunoblot of pro and cleaved CASP11 in uninfected and *E japonica*-infected primary WT and MyD88^-/-^ HCs at 24 hr p.i. **(F, G)** Immunoblot analysis of pro and cleaved Gasdermin (GSDM) D protein normalized to β-actin in uninfected and *E japonica*-infected primary WT and MyD88^-/-^ HCs in the presence/absence of CASP1 and 11 inhibitor at 24 hr p.i. **(H)** LDH percentage release in uninfected and *E japonica*-infected HCs in the presence/absence of MyD88 inhibitor at 24 hr p.i. All results are presented as mean ± SD (*P<0.05, **P<0.01, ***P<0.001) from three independent experiments. ns, not significant.

Our previous studies indicated that MyD88 signaling triggers activation of canonical (CASP1-mediated) and non-canonical (CASP11-mediated) inflammasomes in macrophages in a host-pathogenic mechanism that mediates liver injury (5). We examined whether CASP1 is activated in HCs following *E. japonica* infection, and the role of MyD88 in regulation of HC-specific *Casp1*. *E. japonica* infection of primary WT HCs did not trigger significant upregulation of *Casp1* or *IL-1β* mRNA compared to uninfected WT HCs. However, MyD88 deficiency in primary *E. japonica*-infected MyD88^-/-^ HCs resulted in upregulation of both *Casp1* and *IL-1β* mRNA compared to uninfected primary MyD88^-/-^HCs and infected WT HCs (**Figures 2A–D**). Consistent with mRNA data, immunoblot analysis showed an elevated quantity of cleaved CASPl in *E. japonica*-infected primary MyD88^-/-^ HCs compared to *E. japonica*-infected primary WT HCs and uninfected cells (**Figures 2A, B**). These data suggest that MyD88 inhibits CASPl activation in HCs following *E. japonica* infection. Notably, activation of CASPl was inversely correlated with activation of CASP3 (apoptotic marker), such that *E. japonica*-infected primary MyD88^-/-^ HCs showed lower levels of active CASP3 compared to *E. japonica*-infected primary WT HCs (**Figures 3A, B** **).** These data suggest that MyD88 signaling inhibits CASPl activation, while promoting CASP3 activation in HCs following *E. japonica* infection.

Given our observation of an inverse relationship between CASP1 and CASP3 activation, we hypothesized that active CASP1 in HCs may play a hepatoprotective role via negative regulation of CASP3 activation. To address this possibility, we examined CASP3 activation in *E. japonica*-infected MyD88^-/-^ HCs and control-infected WT-HCs (showing a reduced amount of active CASP1) in the presence of a CASP1 inhibitor. Our findings revealed that the CASP1 inhibitor had no discernible impact on the extent of CASP3 activation in infected WT HCs. However, notably, inhibition of CASP1 resulted in a significant increase in the level of active CASP3 in MyD88^-/-^ HCs (**Figures 3A, B**). This suggests that CASP3 activation in infected HCs is negatively regulated by active CASP1 in the absence of MyD88. In other words, MyD88 promotes CASP3 activation via inhibition of CASP1.

Our prior studies have shown that *E. japonica* infection triggers CASP11 activation in macrophages, which is associated with fatal *E. japonica* infection in mice (9). We, therefore, examined the contribution of MyD88 to CASP11 activation following infection of HCs with *E. japonica*. Infection of WT primary HCs with *E. japonica* increased the mRNA levels of *Casp11* (**Figure 3C**). At the protein level, our investigation revealed that *E. japonica*-infected primary MyD88^-/-^ hepatocytes (HCs) exhibited a decreased quantity of active CASP11 compared to *E. japonica*-infected primary WT HCs (**Figures 3D, E**). This data suggests that MyD88 promotes activation of CASP11. Notably, activation of CASP11 in infected HC cell line correlated with host cell pyroptosis and inflammatory host cell death measured by an increased level of LDH release compared to uninfected cells. On the other hand, treatment of *E. japonica*-infected HC cell line with MyD88 inhibitor attenuated LDH release compared to *E. japonica* infected but untreated HCs (**Figure 3H**). HC cell line cultured with or without MyD88 inhibitor has the same low level of LDH, suggesting that MyD88 inhibitor alone does not have cytotoxic effect (**Figure 3H** **).**

Both active CASP1 and CASP11 play a role in inducing cell death through Gasdermin D cleavage in response to inflammasome activation (30). Given that active CASP1 is higher, and active CASP11 is lower in MyD88^-/-^, we examined Gasdermin D cleavage as a potential mechanism that induces pyroptotic cell death and LDH release. To this end, we infected WT and MyD88^-/-^ HCs with *E. japonica* and examined Gasdermin D cleavage. Our data demonstrate that infection of WT-HCs leads to Gasdermin D cleavage compared to uninfected cells at 24 hr p.i. In contrast, consistent with CASP11 activation, *E. japonica* infection of MyD88^-/-^ HCs did not result in Gasdermin D cleavage at same time point when compared to uninfected MyD88^-/-^ HCs and infected WT HCs. These findings suggest that *E. japonica* induced, MyD88-mediated pyroptosis occurs via Gasdermin D cleavage. To determine whether Gasdermin D cleavage is regulated by CASP1 and/or CASP11, we infected WT- and MyD88^-/-^ HCs in the presence or absence of CASP1 and CASP11 inhibitors. Our data showed that *Ehrlichia*-mediated cleavage of Gasdermin D in WT-HCs is abrogated upon treatment of cells with CASP11, but not CASP1 inhibitor, suggesting that *Ehrlichia*-induced, MyD88-mediated cleavage of Gasdermin D in HCs is CASP11 dependent (**Figures 3F, G**). Taken together, these data further suggest that MyD88 triggers death of HCs via activation of apoptotic CASP3 pathway and non-canonical CASP11-mediated inflammasome pathway, respectively, while inhibiting CASP1 activation.

### MyD88 inhibits HMGB1 cytosolic translocation

**Figure 4:**
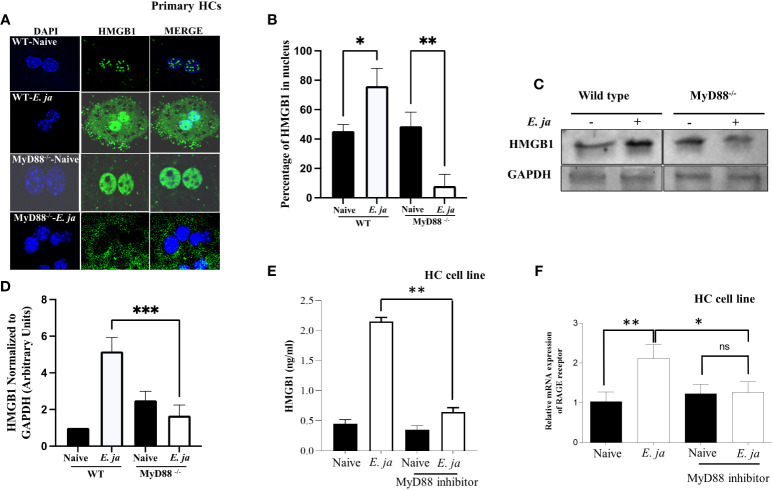
Effects of MyD88 signaling on HMGB1 nuclear translocation and extracellular secretion. **(A)** Immunofluorescence staining showing HMGB1 accumulation in uninfected and *E japonica*-infected primary WT and MyD88^-/-^ HCs at 24 hr p.i. **(B)** Quantitative analysis showing the percentage of HMGB1 in the nucleus in uninfected and *E japonica*-infected primary WT and MyD88^-/-^ HCs **(C)** Western blot of HMGB1 proteins levels in uninfected and *E japonica*-infected primary WT and MyD88^-/-^ HCs at 24 hr p.i. **(D)** Normalized level of total HMGB1 in relation to GAPDH **(E)** ELISA of HMGB1 levels in the culture supernatant of uninfected and *E japonica*-infected HCs in the presence/absence of MyD88 inhibitor at 24 hr p.i. **(F)** Relative mRNA expression of RAGE receptor in uninfected and *E japonica*-infected HCs in the presence/absence of MyD88 inhibitor at 24 hr p.i. All *in vitro* experiments are done thrice and are independent, represented as Mean ± SD (*P<0.05, **P<0.01). ns, not significant.

HMGB1 is a nuclear protein that acts as a DAMP when translocated to the cytosol. Our recently published study revealed that HMGB1 is secreted by HCs following *E. japonica* infection and that secretion is CASP11-dependent (7). Extracellular HMGB1 is secreted actively or passively following pyroptotic inflammatory cell death. To determine the contribution of MyD88 to HMGB1 secretion, we first measured HMGB1 cytosolic translocation in primary WT and MyD88^-/-^ HCs at 24 hr after *E. japonica* infection using immunofluorescence staining. Our data showed that HMGB1 was detected in the nucleus and cytosol of *E. japonica*-infected WT-HCs with a higher nuclear than cytoplasmic expression or localization compared to uninfected WT HCs. In contrast, MyD88^-/-^ HCs exhibited significant cytosolic translocation of HMGB1 (**Figures 4A, B**) with minimal nuclear localization compared to uninfected MyD88^-/-^ HCs as well as infected WT-HCs. The difference in HMGB1 localization between WT and MyD88^-/-^ HCs was associated with a significantly higher protein level of HMGB1 in primary WT-HCs compared to similarly infected MyD88^-/-^ HCs at 24 hr p.i. (**Figures 4C, D**).

Since the total HMGB1 was increased in infected primary WT-HCs, we hypothesized that the small number of cytosolic HMGB1 in primary WT compared to MyD88^-/-^ HCs is due to the secretion of HMGB1 secondary to the activation of CASP11 and pyroptosis. To this end, we measured HMGB1 in cells or the culture supernatant from WT HCs and MyD88^-/-^ HCs 24 hr after *E. japonica* infection. Compared to uninfected WT HCs, *E. japonica*-infected HCs secreted a significantly higher amount of HMGB1 in culture supernatant. Notably, the lack of MyD88 signaling in primary *E. japonica*-infected HCs decreased HMGB1 secretion (**Figure 4E**), despite enhanced cytosolic translocation as shown before. These results could be attributed to decreased CASP11-mediated Gasdermin D cleavage and pyroptotic cell death (**Figures 3D, F, H**) in MyD88^-/-^HCs compared to WT-HCs, which limit or decrease secretion of cytosolic HMGB1.

Studies have shown that extracellular HMGB1 produced by immune or parenchymal cells binds in a paracrine manner to RAGE (the receptor for advanced glycation end products) expressed on adjacent cells (31). Such binding results in arrays of signaling pathways including activation of NF-κb, JAK/STAT and PI3K/AKT among others, which promote inflammatory responses (32) We thus examined whether the excessive dysregulated inflammation seen in the murine model of fatal *E. japonica*-induced liver injury and sepsis is linked to MyD88-mediated expression of RAGE receptor and downstream signals in HCs following binding to extracellular HMGB1. Indeed, *E. japonica* infection of WT-HCs triggered significant upregulation of RAGE mRNA compared to uninfected cells. In contrast, blocking MyD88 signaling in HC cell line significantly abrogated RAGE mRNA expression when compared to infected/untreated HCs (**Figure 4F**). Together, these data suggest that RAGE expression on HCs following *Ehrlichia* infection is MyD88 dependent. Additionally, since MyD88 deficient cells fail to adequately secrete HMGB1, these results indicate a possible positive feedback loop between HMGB1 secretion and upregulation of RAGE.

### MyD88 mediates the induction of autophagy in HCs

**Figure 5:**
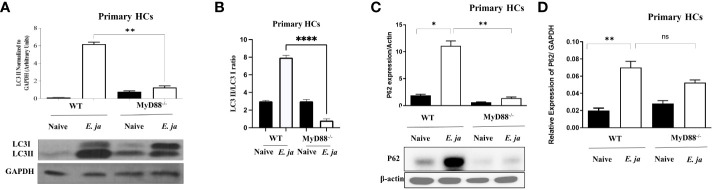
MyD88 mediates the induction of autophagy in HCs. **(A)** Immunoblot of LC3I and LC3II in uninfected and *E japonica*-infected primary WT and MyD88^-/-^ HCs at 24 hr p.i. **(B)** Analyzing LC3II/LC3I ratio in uninfected and *E japonica*-infected WT and MyD88^-/-^ HCs at 24 hr p.i. **(C)** Immunoblot of p62 in uninfected and *E japonica*-infected HCs WT and MyD88^-/-^ HCs at 24 hr p.i. **(D)** Relative mRNA expression of p62 in uninfected and *E japonica*-infected HCs in WT and MyD88^-/-^ HCs at 24 hr p.i. Data is from three independent experiments represented as Mean ± SD (*P<0.05, **P<0.01, ****P<0.0001). ns, not significant.

Prior studies by our lab and other investigators have shown that inflammasome activation in macrophages is negatively regulated by autophagy (5, 33). Therefore, to examine whether MyD88 mediates activation of the non-canonical inflammasome pathway in HCs via regulation of autophagy, we analyzed autophagosome formation. The first step in autophagy involves recruitment of cytosolic LC3 to the phagophore (34). Recruitment of non-lipidated LC3 (LC3I) to autophagosomes involves proteolytic cleavage and lipidation, resulting in LC3II. To investigate this, we infected primary WT HCs and MyD88^-/-^ HCs with *E. japonica* at MOI of 5 and measured autophagy induction by immunoblotting at 24 hr p.i. Immunoblotting analysis demonstrated a significant increase in LC3II quantity, normalized to GAPDH, in *E. japonica*-infected WT HCs compared to uninfected WT HCs, with a 6-fold higher amount of LC3II in WT HCs infected with *E. japonica*. This indicated a higher LC3II quantity and autophagosome formation after *E. japonica* infection in MyD88 sufficient primary HCs. However, the amount of LC3II in *E. japonica*-infected MyD88^-/-^ was lowered compared to infected MyD88 sufficient primary HCs cells. (**Figure 5A**). To validate above results, we analyzed LC3 II/LC3 I ratio and our results demonstrated a significant increase in LC3 II/LC3 I ratio, in *E. japonica*-infected WT HCs compared to uninfected WT HCs, with a 3.2-fold higher ratio in WT HCs infected with *E. japonica*. This indicated a higher LC3 I conversion and autophagosome formation after *E. japonica* infection in MyD88 sufficient primary HCs. Our data also showed that this conversion in *E. japonica*-infected MyD88^-/-^ was lowered compared to infected MyD88 sufficient primary HCs cells. (**Figure 5B**). The elevated level of LC3II in *E. japonica*-infected HCs could be due to blocking autophagy flux. To evaluate autophagy flux in primary HCs, we analyzed the level of p62, a selective autophagy adaptor that binds to ubiquitinated proteins and damaged organelles to traffic them to autophagosome-lysosomal compartments for degradation. The level of p62 is inversely correlated with the autophagic flux and activity. Our results showed a significant accumulation of P62 in *E. japonica*-infected primary WT HCs, suggesting a block of autophagy flux. Notably, lack of MYD88 resulted in decreased accumulation of P62 as evidenced by decreased level of P62 in *E. japonica*-infected MyD88-/- primary HCs compared to *E. japonica*-infected WT HCs (**Figure 5C**). Together, these data suggest that MyD88 signaling in HCs promotes autophagy induction, while blocking autophagy flux during *E. japonica* infection.

### MyD88 compensates for the inhibition of autophagy and mTORC1 activation in HCs

**Figure 6:**
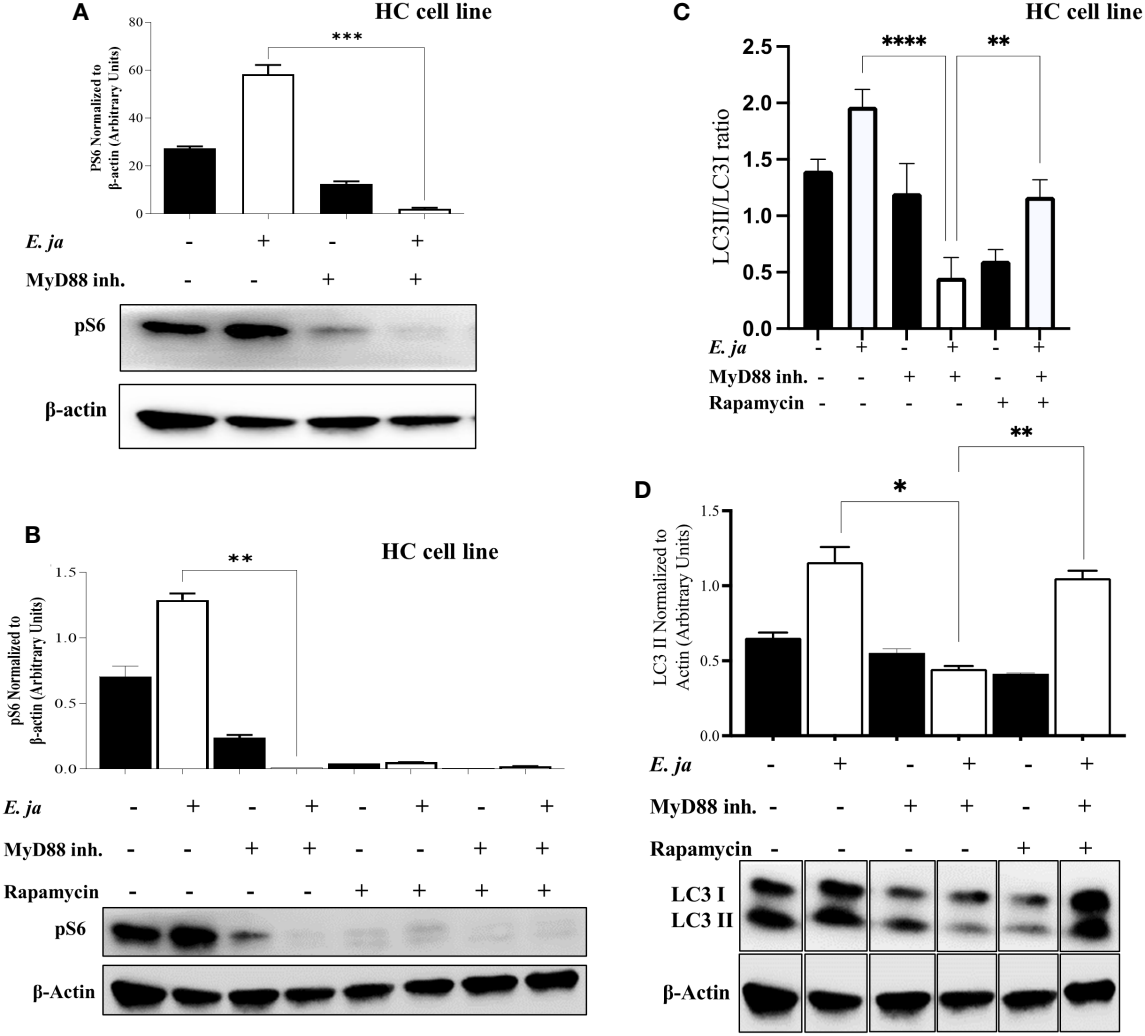
MyD88 compensates for the inhibition of autophagy and mTORC1 activation in HCs. **(A)** Immunoblot of pS6 in uninfected and *E japonica*-infected HCs in the presence/absence of MyD88 inhibitor at 24 hr p.i. **(B)** Immunoblot of pS6 activation in uninfected and *E japonica*-infected HCs with or without MyD88 inhibitor and with or without rapamycin treatment at 24 hr p.i. **(C, D)** Immunoblot of LC3I and LC3II in uninfected and *E.japonica*-infected HCs with/without MyD88 inhibitor and rapamycin at 24 hr p.i. Data is from three independent experiments represented as Mean ± SD (*P<0.05, **P<0.01, ***P<0.001).

mTORC1, a mammalian target of rapamycin complex 1, is a protein complex that functions as a nutrient/energy/redox sensor and controls protein synthesis. We previously reported that MyD88 mTORC1 activation negatively regulates autophagy in macrophages during *E. japonica* infection (5). Therefore, we examined the hypothesis that MyD88 promotes autophagy via inhibition of mTORC1 activation in *E. japonica*-infected HCs. We examined mTORC1 activation by measuring phosphorylation of its downstream target ribosomal protein S6 (pS6). Surprisingly, *E. japonica* infection of untreated HC cell line induced activation of mTORC1 as evidenced by a higher level of quantity of phosphorylated S6 (PS6) downstream target of mTORC1 in these cells compared to uninfected cells. *E. japonica*-infected HC cell line treated with MyD88 inhibitor had a lower level of pS6 at 24 hr p.i. compared to untreated but infected cells or uninfected HCs (**Figure 6A**), suggesting that MyD88 signaling induces mTORC1 activation in HCs following *E. japonica* infection. This data is surprising based on the known function of mTORC1 as negative regulator of autophagy. To directly examine the link between MyD88-mTORC1-autophagy axis in *E. japonica*-infected HCs, we measured LC3II protein in *E. japonica*-infected WT and MyD88 inhibitor treated HC cell line upon further addition of rapamycin (mTORC1 inhibitor) at 24 hr p.i. Rapamycin pre-treatment of uninfected or *E. japonica* infected HC cell line that are either treated with MyD88 inhibitor (i.e. MyD88 deficient cells) or left untreated resulted in blocking mTORC1 activation (**Figures 6B, C**). Consistent with findings in **Figure 5A**, lack of MyD88 signals in untreated but *E. japonica*-infected cells attenuate LC3II level. Paradoxically, inhibition of mTORC1 activation in untreated *E. japonica*-infected HCs, with intact MyD88 signaling, decreased autophagy induction as measured by decreased LC3II level (**Figure 6C**). These findings were not due to cell death since inhibition of mTORC1 activation, in presence of MyD88 inhibitor that block MyD88 signaling in *E. japonica*-infected HCs enhanced induction of autophagy (**Figure 6C**). Thus, enhanced autophagy upon blocking mTORC1 in infected HCs is opposed by MyD88 signaling. Together, these data suggest that MyD88, not only promotes autophagy induction in HCs following *E. japonica* infection, but also reverse or counter regulate the inhibition of autophagy induction by mTORC1 in HCs.

### MyD88 mediates secretion of inflammatory molecules from HCs following virulent *E. japonica* infection

Our murine model of fatal ehrlichiosis demonstrated a strong link between MyD88 signaling and cytokine and chemokine storm in a murine model of fatal ehrlichiosis caused by systemic *E. japonica* infection. To determine the contribution of MyD88 signaling to *E. japonica*-induced inflammation in hepatocytes, we examined the secretome of *E. japonica*-infected HC cell line cultured with or without MyD88 inhibitor. *E. japonica* infection of untreated HCs significantly increased the levels of IL-6, which was significantly attenuated in infected cells treated with an inhibitor, suggesting that IL-6 secretion is MyD88-dependent (**Supplementary Figure 3A**). Similarly, *E. japonica* infection led to a higher secretion of GM-CSF (**Supplementary Figure 3C**) by untreated HCs compared to uninfected controls. However, this effect was MyD88-independent. GM-CSF recruit neutrophils to the liver, where they mediate tissue injury during fatal *E. japonica* infection in mice (7, 35). Notably, *E. japonica* infection inhibited secretion of several pro-inflammatory cytokines and chemokines such as IL-12p70, IFN gamma, IL-12p40, TNF receptor II (TNFRII), IL-17A, and leptin (**Supplementary Figures 3D–I**). However, there is no change in M-CSF (**Supplementary Figures 3A, B**). Also, molecules such as TIMP-1, KC (CXCL1), and MIP-1 alpha (CCL3) (**Supplementary Figure 3M**) showed inhibition upon *E. japonica*- infection in MyD88-independent manner. The levels of MIP-1 gamma showed an increase upon *E. japonica*-infection compared to uninfected controls. Although, this effect was MyD88 independent (**Supplementary Figure 3M**). Levels of other molecules such as MCP-1 (CCL2), eotaxin-2 (CCL24), TECK (CCL25), BLC (CXCL13), CXCL1, and Fractalkine (TNFSF8) (**Supplementary Figures 3L–P**) showed minimal change or no change. Other inflammatory chemokines and cytokines did not exhibit any statistically significant differences between untreated *E. japonica*-infected HCs or pretreated with MyD88 inhibitor (**Supplementary Figures 3A–T**).

## Discussion

HME is an emerging tick-borne infectious disease that initially presents with liver dysfunction, hepatic steatosis, and liver damage, resulting in sepsis and fatal multi-organ failure (36, 37). We recently showed that MyD88-deficient mice are partially resistant to lethal *E. japonica* infection, and that MyD88 signaling in macrophages triggers deleterious inflammasome activation in macrophages via blocking autophagy induction and flux (5). In this study, we demonstrate that MyD88 signaling in HCs plays a dual role in the pathogenesis of HME as it also mediates liver immunopathology and cell death, while controlling bacterial replication. Our data showed that MyD88 signaling in HCs triggers CASP11 and CASP3 activation, two major pathways in inflammatory host cell death (pyroptosis and apoptosis, respectively). Interestingly, although *E. japonica* infection triggers activation of CASP1 in HCs, MyD88 signaling suppresses the level of CASP1 activation (**Figures 2A–D**), a key caspase that mediates the cleavage of inflammasome-dependent cytokines IL-1β (**Figure 2D**) and IL-18 (5). These data explain our current and previous findings showing that HCs are not the primary source of IL-1β and IL-18 in early infection (5, 7). These data are distinct from macrophages as CASP1 activation in these cells leads to secretion of IL-1β (7). The exact mechanism by which MyD88 inhibits the activation of CASP1 and the canonical inflammasome pathway is still unclear and will be investigated in future studies. Nevertheless, these data raise questions regarding the biological function of MyD88-mediated inhibition of CASP1 in HCs during fatal *E. japonica* infection. Our data suggest that CASP1 plays a hepatoprotective role. In support of this conclusion, we showed here that CASP1 negatively regulate CASP3 activation (**Figures 3A, B**), a key inducer of apoptotic cell death. Further, we previously showed that *Casp1* knockout mice are highly susceptible to fatal *E. japonica* infection as evidenced by the development of extensive liver damage, higher bacterial burden in the liver and early death compared to WT mice (9). Further, our findings are consistent with other studies showing an increased level of active CASP1 but decreased quantity of active CASP3 in patients who survived sepsis compared with non-survivors (38). Another study showed that *Casp1* deficient macrophages infected with *Francisella*, express elevated levels of active CASP3 and undergo apoptosis suggesting a link between CASP1 and CASP3 (39). In hemorrhagic shock model, *Casp1* ^-/-^ deficiency mice developed systemic inflammation and liver damage further suggesting a hepatoprotective role of CASP1 (40). Together, our data suggests that CASP1 may play a different role in HCs compared to immune cells.

CASP11 activation accounts for deleterious activation of non-canonical inflammasome pathway during fatal ehrlichiosis as suggested by our previous studies (7, 9). Our data show that *E. japonica* infection leads to MyD88-mediated, CASP11-dependent cleavage of Gasdermin D (**Figure 3F**). Activation of CASP11 in macrophages not only induces cell death, but also secretion of HMGB1 via cleavage of Gasdermin D (7). We recently showed that activation of CASP11 in *E. japonica*-infected primary WT-HCs causes secretion of HMGB1, and induction of inflammatory cell death known as pyroptosis (7). In this study, we showed that HMGB1 secretion from *E. japonica* infected HCs is MyD88 dependent. The correlation between MyD88-mediated HMGB1 secretion, CASP11 activation and Gasdermin D cleavage, suggest that MyD88 may trigger HMGB1 secretion via promoting CASP11-mediated Gasdermin D cleavage. Extracellular HMGB1 secreted from infected HCs is a danger signal that could amplify inflammatory responses, apoptosis as well as CASP1 activation in adjacent uninfected cells via binding to TLR4/TLR2 and RAGE receptors, respectively in a paracrine manner (41, 42). Surprisingly, our data suggest a paradoxical relationship between extracellular HMGB1, RAGE and IL-1β. Although HMGB1 secretion and elevated RAGE levels are observed in *E. japonica* infection (**Figures 4E, F**), IL-1β levels in wildtype HCs are not increased. This is paradoxical to our prior finding in macrophages, where *E. japonica* infection leads to activation of CASP1, release of HMGB1 and an increase in IL-1β. This discrepancy might be due to compensatory or alternative signaling pathways that could underlie the observed HMGB1 secretion but lack of IL-1β. One potential mechanism could be mediated by type I IFN and IFNAR pathways. Autocrine and paracrine IFN-I response and IFNAR signaling in HCs following lethal *E. japonica* infection of mice is deleterious and mediates immunopathology and fatal disease by promoting activation of canonical and non-canonical inflammasomes pathways as well as enhancing bacterial survival and/or replication in HCs (43) (7),. Thus, it is possible that MyD88 suppresses or attenuates autocrine IFNAR-mediated CASP1 activation and IL-1β secretion in HCs to prevent excessive inflammation. The negative regulation of IFN-I response by MyD88 in HC is consistent with other studies showing that MYD88 serves as a negative regulator of the IFN-I response in dendritic cells (44, 45).

How MyD88 triggers activation of CASP11 remains elusive. In macrophages, MyD88 signaling following *E. japonica* infection triggered mitochondrial dysfunction via blocking autophagy flux and mitophagy (5, 11). In this study, we showed that MyD88 also blocks autophagy flux in HCs (**Figure 5C**), which could lead to the accumulation of DAMPS. Consistent with other studies, it is possible that mitochondrial DAMPs (e.g., mitochondrial DNA, reactive oxygen species and oxidized cardiolipin) generated upon MyD88 signaling in infected HCs could trigger activation of CASP11 (46–48). These mitochondrial DAMPs may also account for upregulation of TLR9 in *E. japonica*-infected HCs (**Supplementary Figures 2A, B**). The increased expression of TLR9 in *E. japonica*-infected primary HCs and cell line is consistent with our recent study demonstrating pathogenic role of TLR9 signaling in fatal ehrlichiosis as it mediates inflammation and liver damage (7).

Recently, we showed that MyD88 signaling in *E. japonica*-infected macrophages inhibits autophagy induction and flux via mTORC1 activation, which led to activation of CASP 1 and CASP11 (5, 12). In contrast, our data show that MyD88 signaling in infected HCs promotes autophagy induction (**Figure 5**) and mTORC1 activation (**Figure 6**), suggesting that mTORC1-independent autophagy. Additionally, our data suggest that MyD88 promotes autophagy by interfering with or suppressing canonical mTORC1-mediated inhibition of autophagy (**Figures 6C, D** **).** Whether these events happened simultaneously or sequentially during the disease process is not completely understood and will be an area of future investigation. However, we argue that MyD88 may induce autophagy in HCs during *Ehrlichia* infection via two sequential processes; an early induction of non-canonical autophagy pathway followed by a late activation of canonical autophagy pathway. The non-canonical pathway utilizes some, but not all, components of the autophagic machinery and is referred to as LC3-associated phagocytosis (LAP). Thus, consistent with other studies, an initial sensing of bacterial or mitochondrial DNA by TLR9 and downstream signals via MyD88 adaptor could trigger formation of LAP (5, 7, 49, 50). This non canonical autophagy is not regulated by mTORC1 but is dependent on production of reactive oxygen species (ROS) (49). Nevertheless, the correlation between enhanced autophagy and lower number of intracellular *Ehrlichia* in WT-HCs compared to MyD88^-/-^ HCs suggested that HC-specific, MyD88-mediated autophagy could be a host-protective mechanism. This contrasts with the findings by others showing that macrophage-specific autophagy promotes survival and replication of *Ehrlichia* as these bacteria capture essential nutrients through this process (51, 52). Based on these data, it is possible that the early MyD88-mediated induction of autophagy via non canonical pathway is followed by MyD88-mediated inhibition of mTORC1 activation, as the key negative regulator of canonical autophagy pathway. Such signaling would then result in a sustained autophagy in HCs that controls bacterial replication at various stages of infection. One caveat to the conclusion that MyD88-mediated autophagy is a host protective mechanism is the finding that while MyD88 blocks autophagy flux (**Figure 5C**), a key process that targets damaged host molecules as well as intracellular pathogens for autophagic degradation. This paradoxical observation could be explained by potential heightened sensitivity of HCs exhibiting excessive autophagy and mitochondrial dysfunction to cell death because of loss of essential cellular functions. Prior studies indicated that autophagy could induce apoptotic cell death presumably via binding of beclin1 (initial protein required for formation of autophagosome to the anti-apoptotic protein Bcl-2, which trigger cell apoptosis (53). *E. japonica* infect and replicate in HCs as we have shown previously and, in this study (7), and **Figure 1**). Since *Ehrlichia* is an obligate intracellular bacterium that require cell survival for their replication, HCs cell death could potentially limit bacterial survival and replication. Notably, electron microscopic analysis of liver sections from patients with sepsis demonstrated a reproducible pattern of mitochondrial injury and a significant increase in autophagic vacuolization in HCs compared to patients with elective liver biopsies. These autophagic vacuoles in HCs were occurring in cells that were irreversibly committed to cell death in septic patients (54).

**Figure 7:**
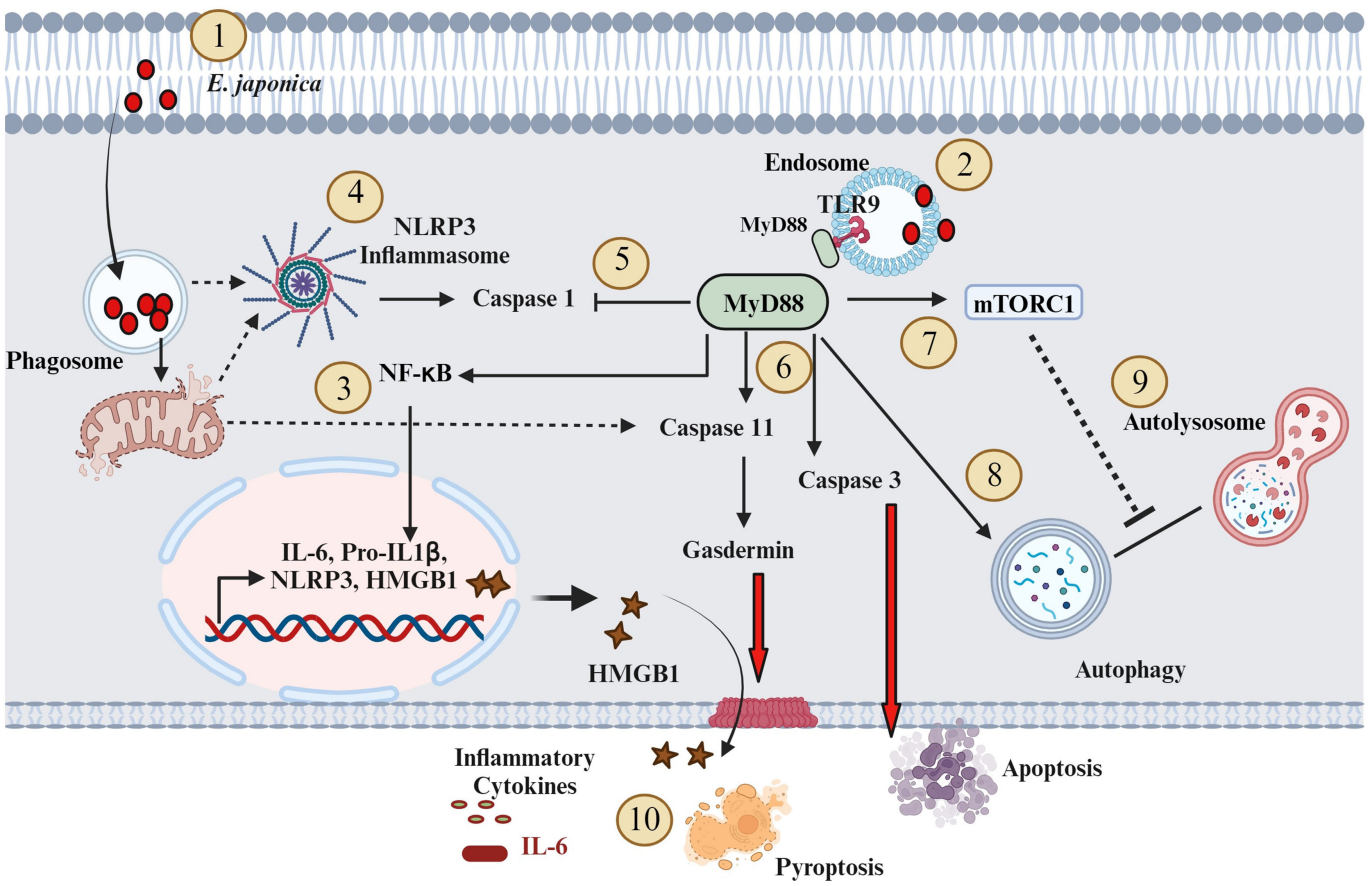
Proposed model of the role of MyD88 in the pathogenesis of *Ehrlichia*-induced liver injury (1). *E. japonica* enters host cells (HCs) and residing within the Phagosome (2); *E. japonica* entry leads to the induction of TLR9 and MyD88 (3); This activation of TLR9 and MyD88 triggers the activation of NF-kB, resulting in the upregulation of inflammatory genes like IL-6, IL-1β, NLRP3, and HMGB1 (4); *E. japonica* triggers mitochondrial damage as suggested by our prior studies. Such mitochondrial dysfunction may result in the release of mitochondrial DAMPS that triggers activation of NLRP3 inflammasome complex. NLRP3 activation trigger cleavage/activation of CASP1 (5); On the other hand, MyD88 signals negatively regulate CASP1 activation, which accounts for lack of secretion of biologically active IL-1β by infected HCs (6); MyD88 activates CASP11 and CASP3. CASP3 causes apoptotic cell death, while activated CASP11 induces Gasdermin D, leading to pyroptosis and release of HMGB1 (7); MyD88 also activates mTORC1 (8); MyD88 also promotes an early induction of mTORC1-independent non-canonical autophagy (9); MyD88 also blocks autophagy flux via mechanisms that may include activation of mTORC1; and (10) The above downstream signaling pathways mediated by MyD88 in HC following infection with virulent *E. japonica* result in host-pathogenic response including inflammation and apoptotic and pyroptotic cell death. Solid arrows indicate established mechanisms by data in this study as well as our previous studies. The dotted arrows indicate hypothetical mechanisms.

In conclusion, we proposed a novel hepatocyte-specific, pathogenic, and protective roles of MyD88 signaling in the pathophysiology of acute liver injury during fatal ehrlichiosis (**Figure 7**). The pathogenic role of hepatocyte-specific MyD88 signaling is mediated by activation of CASP3 as well as CASP 11-Gasdermin D axis (non-canonical inflammasome pathway) promoting apoptotic and pyroptotic cell death as well as HMGB1 release and inflammation. On the other hand, the protective role of hepatocyte specific MyD88 involves controlling bacterial replication. Whether MyD88-mediated control of bacterial replication in HCs involves induction of non-apoptotic (i.e., autophagic) programmed cell death induction that restrict survival and replication of *Ehrlichia* in non-myeloid cells remain unknown. Although we used primary hepatocytes in this study to mimic the *in vivo* system, the *in vitro* culture system remains a limitation. Future studies using knockout mice with hepatocyte-specific deletion of MyD88 (Myd88^Δ^Hep) would overcome this limitation. Further, the protective role of MyD88 in ehrlichiosis was only examined here in the context of infection with highly virulent *E. japonica* species that cause lethal infection in mice. Future studies employing infection with mildly virulent *E. muris* that cause mild/non-lethal ehrlichiosis in mice would provide more insight into the anti-bacterial protective function of MyD88. In that regard, we also aim to unravel the mechanisms through which *E. japonica* induces autophagy in host cells, encompassing both mTORC1-dependent and mTORC1-independent pathways. Finally, future studies will also explore the unconventional hepatoprotective role of the CASP1 activation/canonical inflammasome pathway in hepatocytes during *E. japonica* infection.

## Data availability statement

The datasets presented in this study can be found in online repositories. The names of the repository/repositories and accession number(s) can be found in the article/**Supplementary Material**.

## Ethics statement

The animal experiments conducted in the study was approved by the University of Illinois at Chicago Animal Care and Committee as well as the University of Pittsburgh Animal Research Protection Committee. The Animal study was conducted in accordance with the local legislation and institutional requirements. All animal experiments were performed according to the guidelines of the American Association for the Assessment and Accreditation of Lab Animal Care (AAALAC).

## Author contributions

NI did the experimental design. OT, MA, AS, MK, IA, HE performed and interpreted the experiments. OT wrote the manuscript. NI provided supervision, funding, resources, interpreted results, and reviewed the manuscript. All authors contributed to the article and approved the submitted version.

## References

[1] Rikihisa Y. Anaplasma phagocytophilum and *Ehrlichia* chaffeensis: subversive manipulators of host cells. Nat Rev Microbiol (2010) 8(5):328–39. doi: 10.1038/nrmicro2318 20372158

[2] McBride JW Walker DH. Progress and obstacles in vaccine development for the ehrlichioses. Expert Rev Vaccines (2010) 9(9):1071–82. doi: 10.1586/erv.10.93 PMC2951016 20822349

[3] Moumene A Meyer DF. *Ehrlichia*’s molecular tricks to manipulate their host cells. Microbes infection (2016) 18(3):172–9. doi: 10.1016/j.micinf.2015.11.001 26617397

[4] McBride JW Walker DH. Molecular and cellular pathobiology of Ehrlichia infection: targets for new therapeutics and immunomodulation strategies. Expert Rev Mol Med (2011) 13:13. e3. doi: 10.1017/S1462399410001730 PMC3767467 21276277

[5] Kader M Alaoui-El-Azher M Vorhauer J Kode BB Wells JZ Stolz D. MyD88-dependent inflammasome activation and autophagy inhibition contributes to *Ehrlichia*-induced liver injury and toxic shock. PloS Pathog (2017) 13(10):e1006644. doi: 10.1371/journal.ppat.1006644 29049365 PMC5663626

[6] Tominello TR Oliveira ER Hussain SS Elfert A Wells J Golden B. Emerging roles of autophagy and inflammasome in ehrlichiosis. Front Immunol (2019) 10:1011. doi: 10.3389/fimmu.2019.01011 31134081 PMC6517498

[7] Kader M El Andaloussi A Vorhaour J Tamama K Nieto N Scott MJ. Interferon type I regulates inflammasome activation and high mobility group box 1 translocation in hepatocytes during *ehrlichia*-induced acute liver injury. Hepatol Commun (2021) 5(1):33–51. doi: 10.1002/hep4.1608 33437899 PMC7789844

[8] Lin M Xiong Q Chung M Daugherty SC Nagaraj S Sengamalay N. Comparative analysis of genome of *ehrlichia* sp. HF, a model bacterium to study fatal human ehrlichiosis. BMC Genomics (2021) 22(1):11. doi: 10.1186/s12864-020-07309-z 33407096 PMC7789307

[9] Yang Q Stevenson HL Scott MJ Ismail N. Type I interferon contributes to noncanonical inflammasome activation, mediates immunopathology, and impairs protective immunity during fatal infection with lipopolysaccharide-negative *Ehrlichia*e. Am J Pathol (2015) 185(2):446–61. doi: 10.1016/j.ajpath.2014.10.005 PMC4305182 25481711

[10] Voss OH Rahman MS. Rickettsia-host interaction: strategies of intracytosolic host colonization. Pathog Dis (2021) 79(4):ftab015. doi: 10.1093/femspd/ftab015 PMC8023194 33705517

[11] Haloul M Oliveira ER Kader M Wells JZ Tominello TR El Andaloussi A. mTORC1-mediated polarization of M1 macrophages and their accumulation in the liver correlate with immunopathology in fatal ehrlichiosis. Sci Rep (2019) 9(1):1–13. doi: 10.1038/s41598-019-50320-y 31575880 PMC6773708

[12] Vorhauer J Alaoui-El-Azher M Wells A Ismail N. Autophagy and Inflammasome activation triggered by LPS-negative *Ehrlichia* is dependent on both mTOR activation and MyD88 signaling. (2016) J Immunol 196 (1_Supplement): 62:12. doi: 10.4049/jimmunol.196.Supp.62.12

[13] Pedra JH Sutterwala FS Sukumaran B Ogura Y Qian F Montgomery RR. ASC/PYCARD and caspase-1 regulate the IL-18/IFN-γ axis during Anaplasma phagocytophilum infection. J Immunol (2007) 179(7):4783–91. doi: 10.4049/jimmunol.179.7.4783 17878377

[14] Lu B Wang H Andersson U Tracey KJ. Regulation of HMGB1 release by inflammasomes. Protein Cell (2013) 4(3):163–7. doi: 10.1007/s13238-012-2118-2 PMC4533838 23483477

[15] Tang D Kang R Livesey KM Cheh C-W Farkas A Loughran P. Endogenous HMGB1 regulates autophagy. J Cell Biol (2010) 190(5):881–92. doi: 10.1083/jcb.200911078 PMC2935581 20819940

[16] Wang J He G-Z Wang Y-K Zhu Q-K Chen W Guo T. TLR4-HMGB1-, MyD88-and TRIF-dependent signaling in mouse intestinal ischemia/reperfusion injury. World J Gastroenterology: WJG (2015) 21(27):8314. doi: 10.3748/wjg.v21.i27.8314 PMC4507101 26217083

[17] Bafica A Scanga CA Feng CG Leifer C Cheever A Sher A. TLR9 regulates Th1 responses and cooperates with TLR2 in mediating optimal resistance to Mycobacterium tuberculosis. J Exp Med (2005) 202(12):1715–24. doi: 10.1084/jem.20051782 PMC2212963 16365150

[18] Yu M Wang H Ding A Golenbock DT Latz E Czura CJ. HMGB1 signals through toll-like receptor (TLR) 4 and TLR2. Shock (2006) 26(2):174–9. doi: 10.1097/01.shk.0000225404.51320.82 16878026

[19] Wang H Ward MF Sama AE. Targeting HMGB1 in the treatment of sepsis. Expert Opin Ther Targets (2014) 18(3):257–68. doi: 10.1517/14728222.2014.863876 PMC3945414 24392842

[20] Deng M Scott MJ Fan J Billiar TR. Location is the key to function: HMGB1 in sepsis and trauma-induced inflammation. J leukocyte Biol (2019) 106(1):161–9. doi: 10.1002/JLB.3MIR1218-497R PMC6597316 30946496

[21] Cheng Y Wang D Wang B Li H Xiong J Xu S. HMGB1 translocation and release mediate cigarette smoke–induced pulmonary inflammation in mice through a TLR4/MyD88-dependent signaling pathway. Mol Biol Cell (2017) 28(1):201–9. doi: 10.1091/mbc.e16-02-0126 PMC5221624 27807045

[22] Cheng Y Liu Y Wu B Zhang J-Z Gu J Liao Y-L. Proteomic analysis of the *Ehrlichia* chaffeensis phagosome in cultured DH82 cells. PloS One (2014) 9(2):e88461. doi: 10.1371/journal.pone.0088461 24558391 PMC3928192

[23] Perez M Rikihisa Y Wen B. *Ehrlichia* canis-like agent isolated from a man in Venezuela: antigenic and genetic characterization. J Clin Microbiol (1996) 34(9):2133–9. doi: 10.1128/jcm.34.9.2133-2139.1996 PMC229204 8862572

[24] Gaul S Leszczynska A Alegre F Kaufmann B Johnson CD Adams LA. Hepatocyte pyroptosis and release of inflammasome particles induce stellate cell activation and liver fibrosis. J Hepatol (2021) 74(1):156–67. doi: 10.1016/j.jhep.2020.07.041 PMC7749849 32763266

[25] Chen J He J Yang Y Jiang J. An analysis of the expression and function of myeloid differentiation factor 88 in human osteosarcoma. Oncol Lett (2018) 16(4):4929–36. doi: 10.3892/ol.2018.9297 PMC6144908 30250559

[26] Shiratori E Itoh M Tohda S. MYD88 inhibitor ST2825 suppresses the growth of lymphoma and leukaemia cells. Anticancer Res (2017) 37(11):6203–9. doi: 10.21873/anticanres.12070 29061802

[27] Blasius AL Beutler B. Intracellular toll-like receptors. Immunity (2010) 32(3):305–15. doi: 10.1016/j.immuni.2010.03.012 20346772

[28] Kawasaki T Kawai T. Toll-like receptor signaling pathways. Front Immunol (2014) 5:461. doi: 10.3389/fimmu.2014.00461 25309543 PMC4174766

[29] Rikihisa Y Kawahara M Wen B Kociba G Fuerst P Kawamori F. Western immunoblot analysis of Haemobartonella muris and comparison of 16S rRNA gene sequences of H. muris, H. felis, and Eperythrozoon suis. J Clin Microbiol (1997) 35(4):823–9. doi: 10.1128/jcm.35.4.823-829.1997 PMC229683 9157135

[30] Hu Y Jiang Y Li S Ma X Chen M Yang R. The Gasdermin D N-terminal fragment acts as a negative feedback system to inhibit inflammasome-mediated activation of Caspase-1/11. Proc Natl Acad Sci U.S.A. (2022) 119(45):e2210809119. doi: 10.1073/pnas.2210809119 36322773 PMC9659347

[31] Khambu B Yan S Huda N Yin XM. Role of high-mobility group box-1 in liver pathogenesis. Int J Mol Sci (2019) 20(21): 5314. doi: 10.3390/ijms20215314 PMC6862281 31731454

[32] Amornsupak K Thongchot S Thinyakul C Box C Hedayat S Thuwajit P. HMGB1 mediates invasion and PD-L1 expression through RAGE-PI3K/AKT signaling pathway in MDA-MB-231 breast cancer cells. BMC Cancer (2022) 22(1):578. doi: 10.1186/s12885-022-09675-1 35610613 PMC9128129

[33] Paik S Kim JK Silwal P Sasakawa C Jo EK. An update on the regulatory mechanisms of NLRP3 inflammasome activation. Cell Mol Immunol (2021) 18(5):1141–60. doi: 10.1038/s41423-021-00670-3 PMC8093260 33850310

[34] Morel E. Endoplasmic reticulum membrane and contact site dynamics in autophagy regulation and stress response. Front Cell Dev Biol (2020) 8:343. doi: 10.3389/fcell.2020.00343 32548114 PMC7272771

[35] Yang Q Ghose P Ismail N. Neutrophils mediate immunopathology and negatively regulate protective immune responses during fatal bacterial infection-induced toxic shock. Infect Immun (2013) 81(5):1751–63. doi: 10.1128/IAI.01409-12 PMC3647993 23478316

[36] Ismail N McBride JW. Tick-borne emerging infections: ehrlichiosis and anaplasmosis. Clinics Lab Med (2017) 37(2):317–40. doi: 10.1016/j.cll.2017.01.006 28457353

[37] Biggs HM Behravesh CB Bradley KK Dahlgren FS Drexler NA Dumler JS. Diagnosis and management of tickborne rickettsial diseases: rocky mountain spotted fever and other spotted fever group rickettsioses, ehrlichioses, and anaplasmosis - United States. MMWR Recomm Rep (2016) 65(2):1–44. doi: 10.15585/mmwr.rr6502a1 27172113

[38] Aziz M Jacob A Wang P. Revisiting caspases in sepsis. Cell Death Dis (2014) 5(11):e1526. doi: 10.1038/cddis.2014.488 25412304 PMC4260746

[39] Pierini R Juruj C Perret M Jones CL Mangeot P Weiss DS. AIM2/ASC triggers caspase-8-dependent apoptosis in Francisella-infected caspase-1-deficient macrophages. Cell Death Differ (2012) 19(10):1709–21. doi: 10.1038/cdd.2012.51 PMC3438500 22555457

[40] Menzel CL Sun Q Loughran PA Pape HC Billiar TR Scott MJ. Caspase-1 is hepatoprotective during trauma and hemorrhagic shock by reducing liver injury and inflammation. Mol Med (2011) 17(9-10):1031–8. doi: 10.2119/molmed.2011.00015 PMC3188860 21666957

[41] Ismail N Sharma A Soong L Walker DH. Review: protective immunity and immunopathology of ehrlichiosis. Zoonoses (Burlingt) (2022) 2(1):10.15212. doi: 10.15212/ZOONOSES-2022-0009 PMC9300479 35876763

[42] Yang H Wang H Andersson U. Targeting inflammation driven by HMGB1. Front Immunol (2020) 11:484. doi: 10.3389/fimmu.2020.00484 32265930 PMC7099994

[43] Zhang Y Thai V McCabe A Jones M MacNamara KC. Type I interferons promote severe disease in a mouse model of lethal ehrlichiosis. Infect Immun (2014) 82(4):1698–709. doi: 10.1128/IAI.01564-13 PMC3993392 24491580

[44] Mizraji G Nassar M Segev H Sharawi H Eli-Berchoer L Capucha T. Porphyromonas gingivalis Promotes Unrestrained Type I Interferon Production by Dysregulating TAM Signaling via MYD88 Degradation. Cell Rep (2017) 18(2):419–31. doi: 10.1016/j.celrep.2016.12.047 28076786

[45] Yu X Du Y Cai C Cai B Zhu M Xing C. Inflammasome activation negatively regulates MyD88-IRF7 type I IFN signaling and anti-malaria immunity. Nat Commun (2018) 9(1):4964. doi: 10.1038/s41467-018-07384-7 30470758 PMC6251914

[46] Santoni G Cardinali C Morelli MB Santoni M Nabissi M Amantini C. Danger-and pathogen-associated molecular patterns recognition by pattern-recognition receptors and ion channels of the transient receptor potential family triggers the inflammasome activation in immune cells and sensory neurons. J Neuroinflamm (2015) 12(1):1–10. doi: 10.1186/s12974-015-0239-2 PMC4322456 25644504

[47] Nakahira K Hisata S Choi AM. The roles of mitochondrial damage-associated molecular patterns in diseases. Antioxid Redox Signal (2015) 23(17):1329–50. doi: 10.1089/ars.2015.6407 PMC4685486 26067258

[48] Marchi S Guilbaud E Tait SWG Yamazaki T Galluzzi L. Mitochondrial control of inflammation. Nat Rev Immunol (2023) 23(3):159–73. doi: 10.1038/s41577-022-00760-x PMC9310369 35879417

[49] Heckmann BL Boada-Romero E Cunha LD Magne J Green DR. LC3-associated phagocytosis and inflammation. J Mol Biol (2017) 429(23):3561–76. doi: 10.1016/j.jmb.2017.08.012 PMC5743439 28847720

[50] Henault J Martinez J Riggs JM Tian J Mehta P Clarke L. Noncanonical autophagy is required for type I interferon secretion in response to DNA-immune complexes. Immunity (2012) 37(6):986–97. doi: 10.1016/j.immuni.2012.09.014 PMC3786711 23219390

[51] Lin M Liu H Xiong Q Niu H Cheng Z Yamamoto A. *Ehrlichia* secretes Etf-1 to induce autophagy and capture nutrients for its growth through RAB5 and class III phosphatidylinositol 3-kinase. Autophagy (2016) 12(11):2145–66. doi: 10.1080/15548627.2016.1217369 PMC5103349 27541856

[52] Rikihisa Y. The “Biological weapons” of *ehrlichia* chaffeensis: novel molecules and mechanisms to subjugate host cells. Front Cell Infect Microbiol (2021) 11:830180. doi: 10.3389/fcimb.2021.830180 35155275 PMC8834651

[53] Maejima Y Isobe M Sadoshima J. Regulation of autophagy by Beclin 1 in the heart. J Mol Cell Cardiol (2016) 95:19–25. doi: 10.1016/j.yjmcc.2015.10.032 26546165 PMC4861696

[54] Watanabe E Muenzer JT Hawkins WG Davis CG Dixon DJ McDunn JE. Sepsis induces extensive autophagic vacuolization in hepatocytes: a clinical and laboratory-based study. Lab Invest (2009) 89(5):549–61. doi: 10.1038/labinvest.2009.8 PMC3822608 19188912

