# Supplementary figures and images for: Hepatocyte-specific regulation of autophagy and inflammasome activation via MyD88 during lethal Ehrlichia infection
Source: Front Immunol. 2023 Nov 7;14:1212167. doi: 10.3389/fimmu.2023.1212167 (PMC10662044; doi:10.3389/fimmu.2023.1212167)

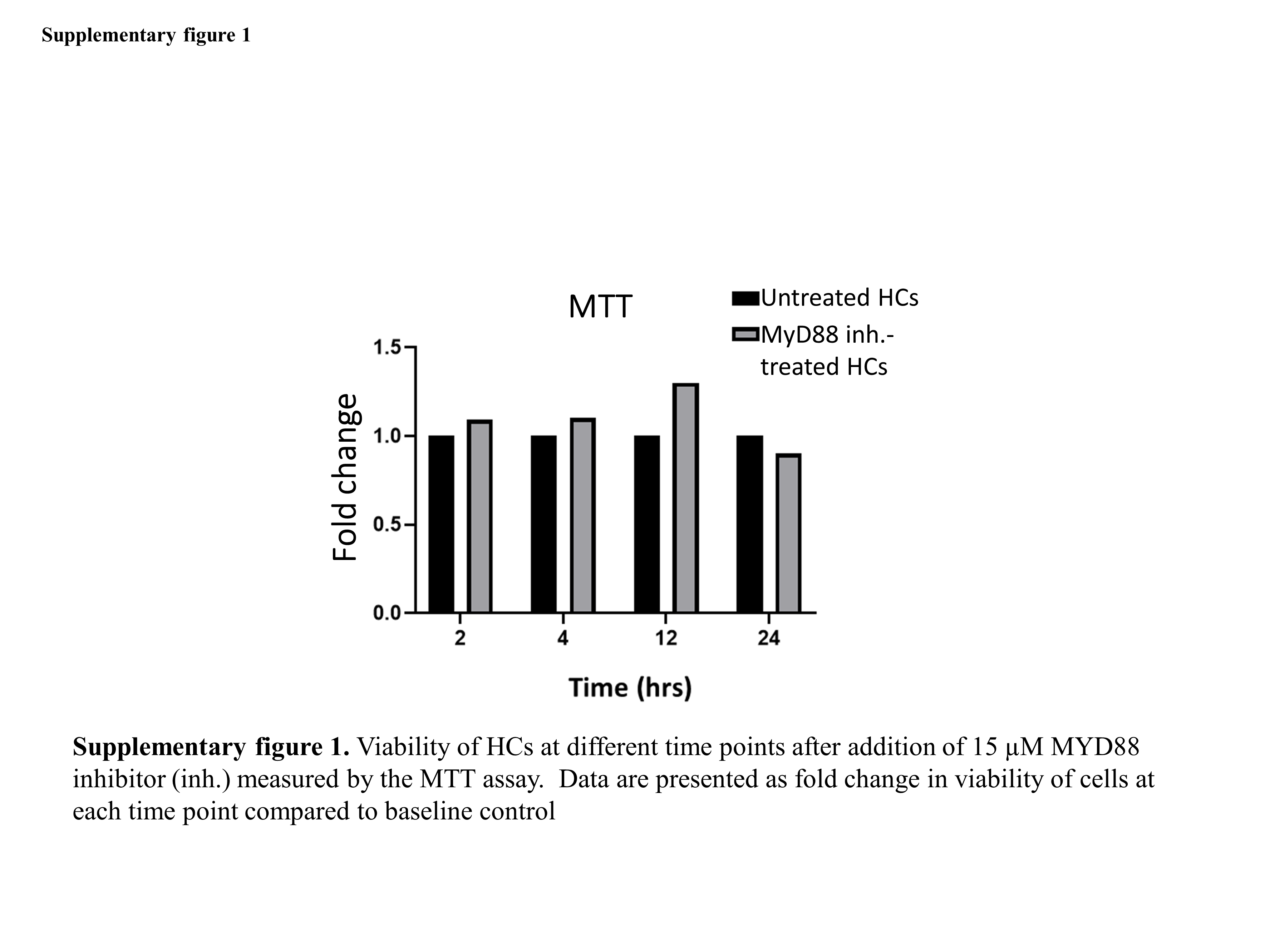

Supplement: Supplementary file 1 [file Image_1.tif]

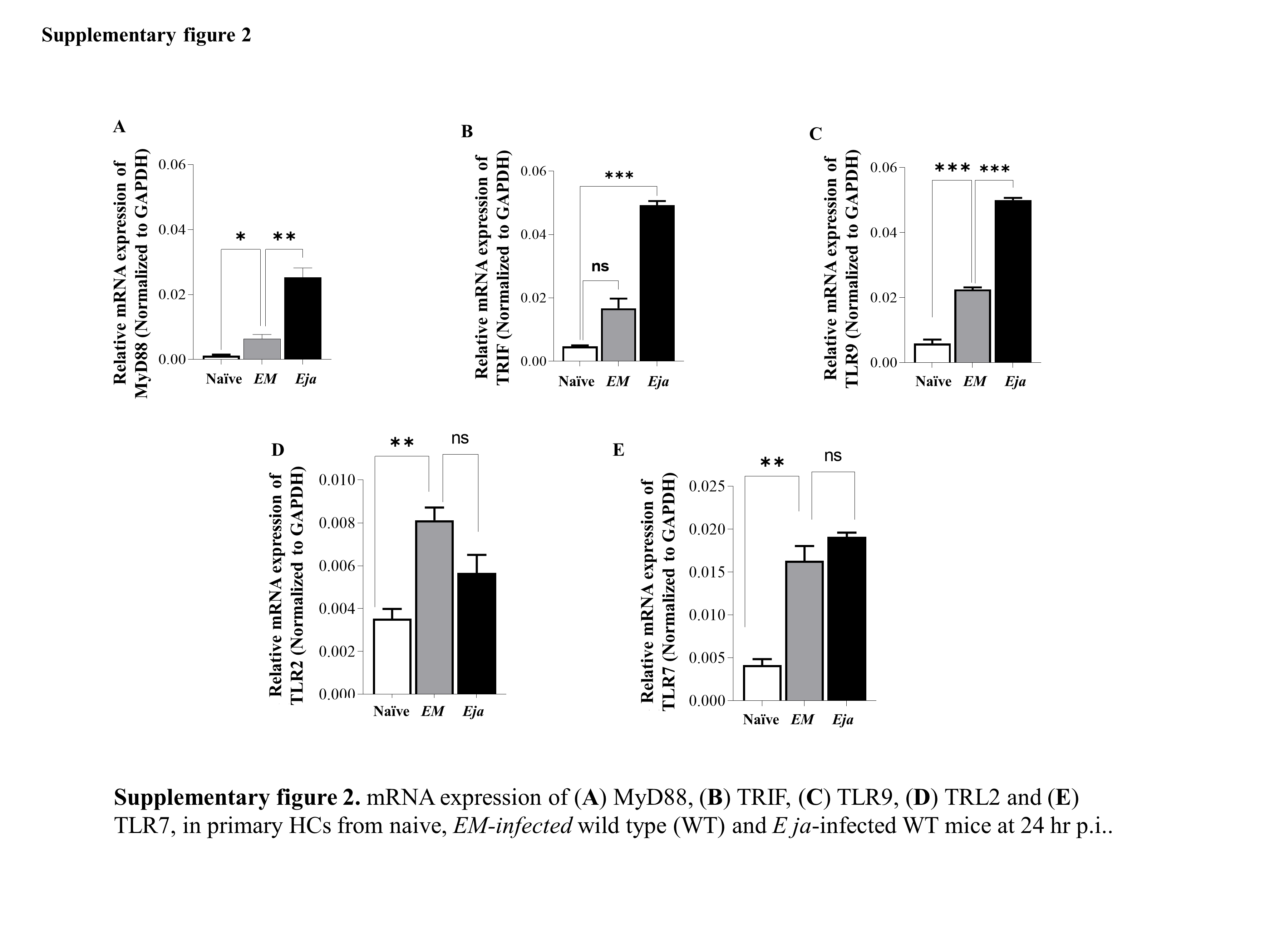

Supplement: Supplementary file 2 [file Image_2.tif]

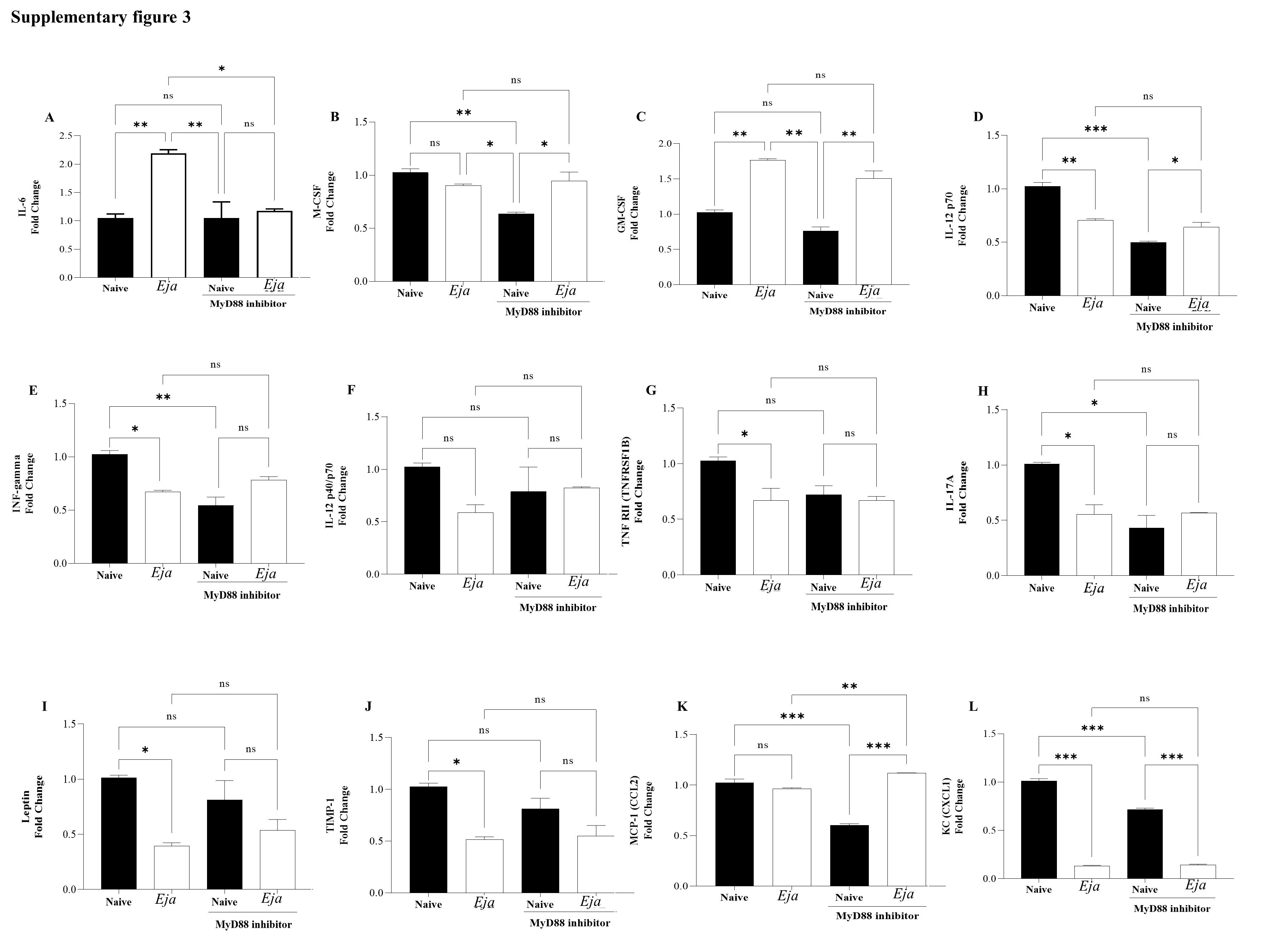

Supplement: Supplementary file 3 [file Image_3.tif]

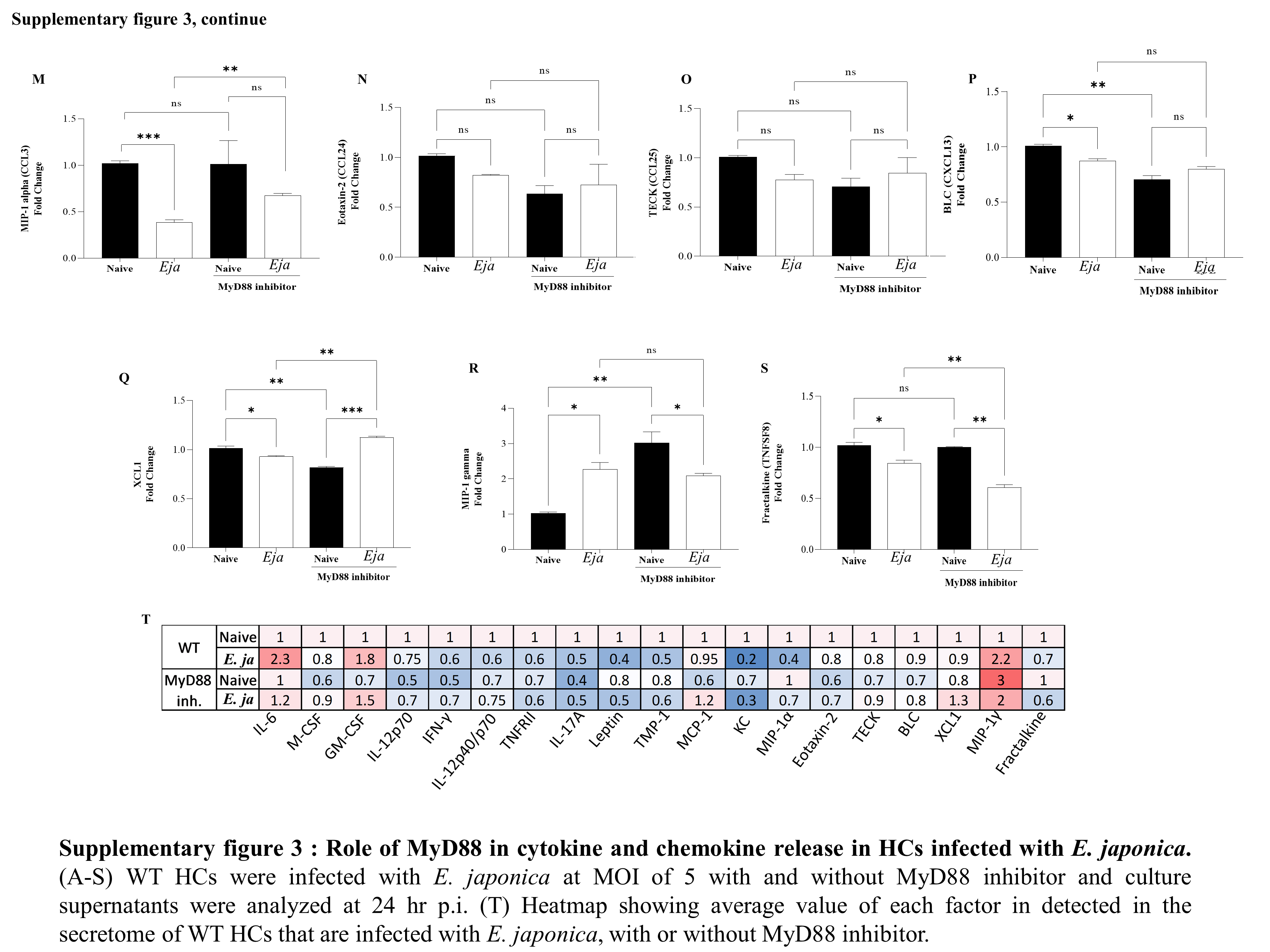

Supplement: Supplementary file 4 [file Image_4.tif]

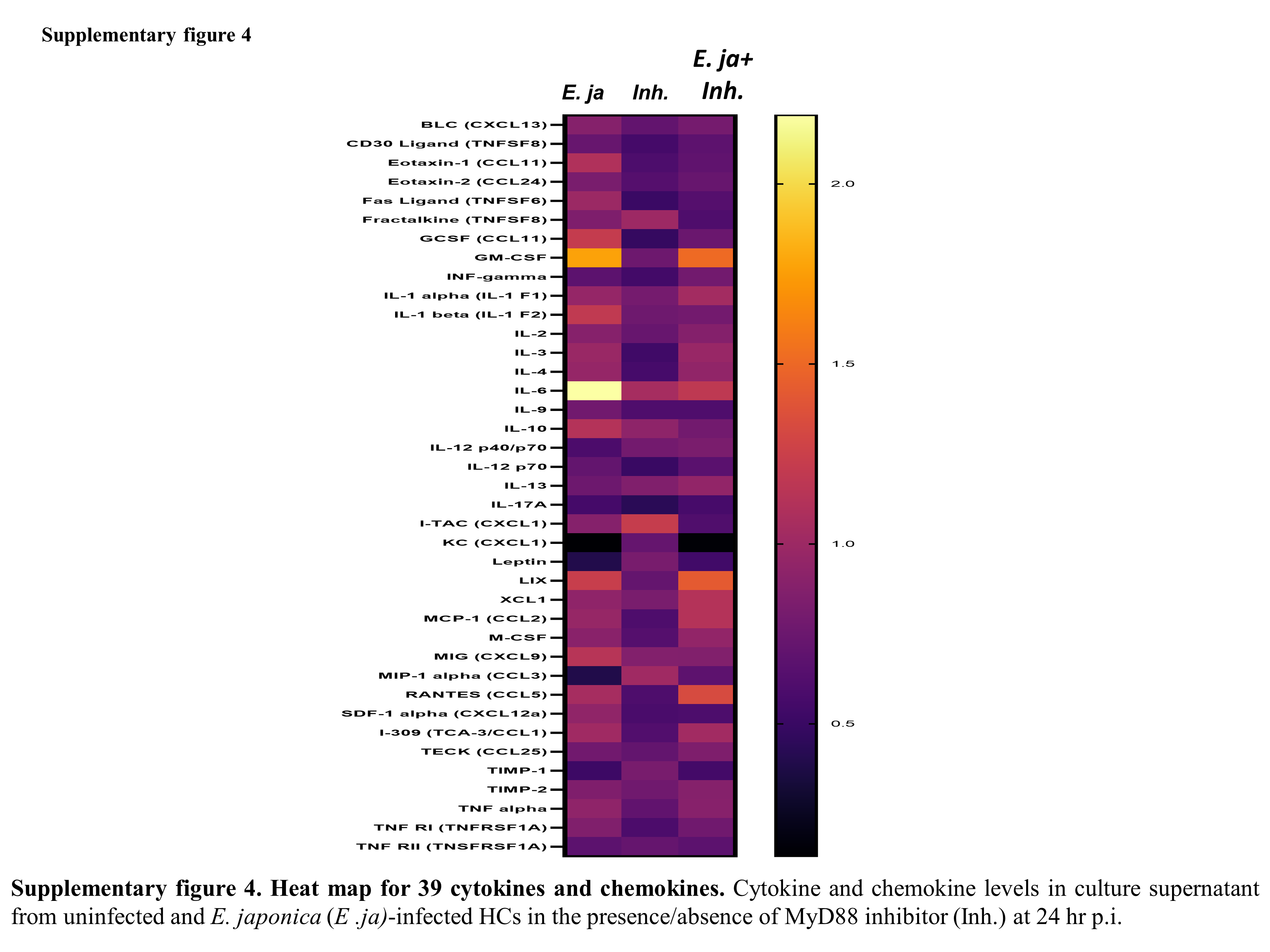

Supplement: Supplementary file 5 [file Image_5.tif]

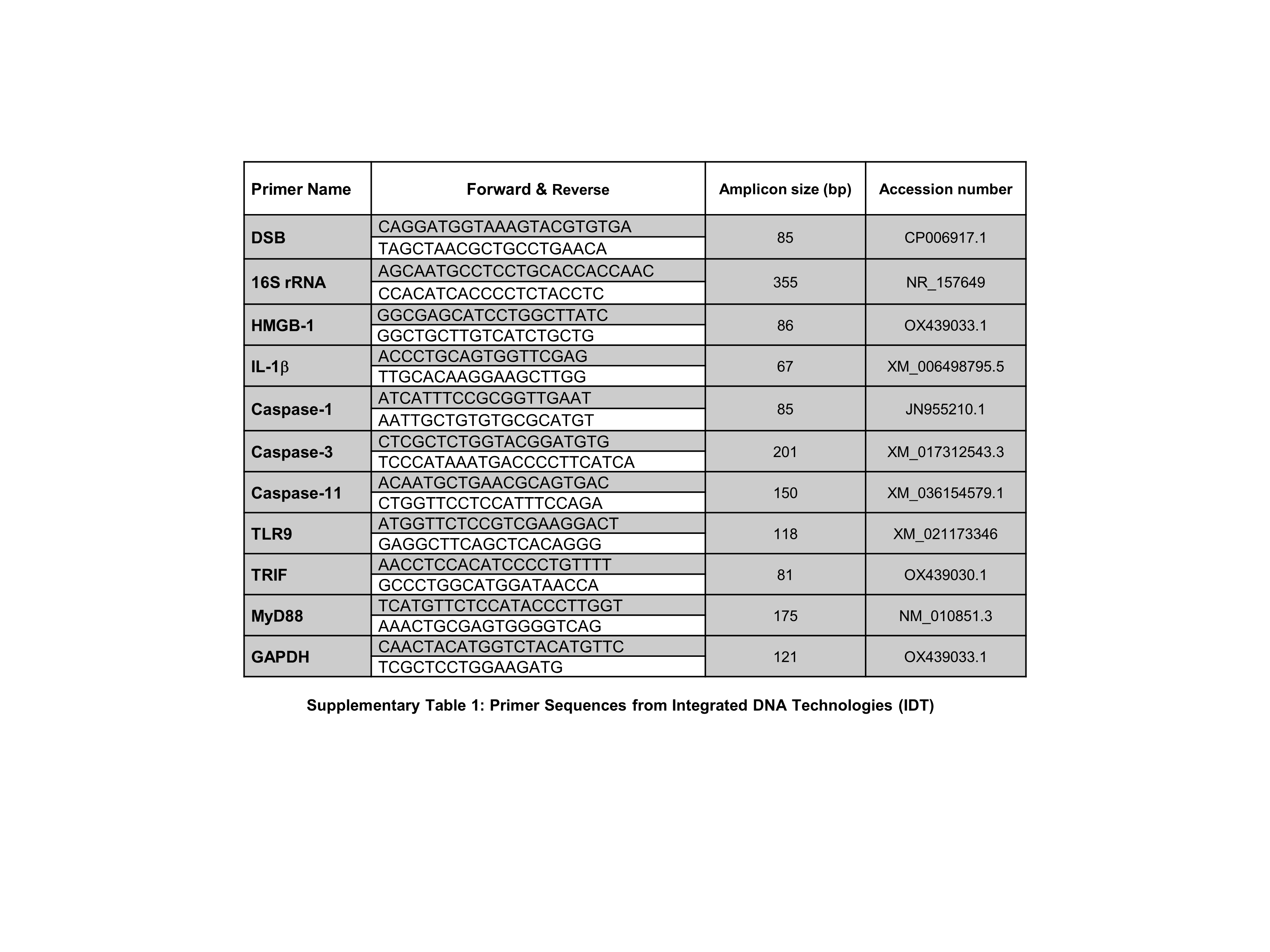

Supplement: Supplementary file 6 [file Image_6.tif]
